# Single-Centre Retrospective Audit of Clostridium difficile Infections Post Ileostomy Reversal

**DOI:** 10.7759/cureus.51674

**Published:** 2024-01-04

**Authors:** Daniel Jia Wei Lee, Mohammed Faisal Bin Abdur Raheem, Andrew Coveney

**Affiliations:** 1 General Surgery, Sir Charles Gairdner Hospital, Perth, AUS

**Keywords:** clinical audit, retrospective cohort study, risk factors, clostridium difficile infection, ileostomy reversal

## Abstract

Background

Clostridium difficile (*C. difficile*) infection can have serious implications on patient outcomes, especially post ileostomy reversal. The symptoms can range from asymptomatic/mild to severe, with significant morbidity or mortality. Thus far, no study has been published to determine the role and impact of preoperative *C. difficile* testing prior to ileostomy reversal. The aim of this audit was to identify risk factors for the development of post-ileostomy reversal *C. difficile* infection and provide further improvements and direction for future research.

Methods

All patients undergoing ileostomy reversal at the General Surgery Department at Sir Charles Gairdner Hospital, a tertiary centre in Perth, Western Australia, were retrospectively identified between January 2019 and June 2021. Demographics and key data points, such as specific types of antibiotic usage, were extracted from patient notes and analysed using IBM SPSS Statistics for Windows, version 27 (released 2020; IBM Corp., Armonk, New York, United States).

Results

Sixty-nine patients were identified in the audit period, with 8.70% of patients testing positive for *C. difficile* infection post ileostomy reversal. At the index ileostomy formation operation, postoperative use of quinolone antibiotics was statistically associated with an increased risk of developing *C. difficile* on ileostomy reversal (odds ratio (OR) = 15.25, confidence interval (CI) 95%, p = 0.035). Intraoperative nitroimidazole use was statistically associated with a reduced risk of *C. difficile* infection on ileostomy reversal (OR = 0.16, CI 95%, p = 0.045). Patients who had diverticulitis as their underlying disease pathology were 10 times more likely to develop *C. difficile* infection post ileostomy reversal, although this finding was not statistically significant in our study.

Conclusion

Several risk factors were identified, such as the use of quinolone antibiotics or having underlying diverticulitis as causes for ileostomy formation. The results from this audit provides further direction in designing further research studies into the role and impact of *C. difficile *testing and treatment in the perioperative period around ileostomy reversal.

## Introduction

A derivative, diverting or loop ileostomy is often conducted as a part of a low/ultra-low colorectal or coloanal anastomoses. It is performed for the treatment of colonic issues, such as colorectal malignancy, inflammatory bowel disease (IBD) (ulcerative colitis, Crohn's disease), perirectal fistulas or diverticulitis. Proximal diversion of stool is often used in higher risk situations, where there are clinical concerns for the development of an anastomotic leak [1]. These ileostomies are reversed approximately six months post formation, pending patients’ general health and condition of the anastomoses (assessed with sigmoidoscopy or computerised tomography with rectal contrast) [2]. The complications of an ileostomy reversal can range from bowel obstruction to perforation, with an associated morbidity of 17.3% [3]. A further lesser reported complication of ileostomy reversal includes *Clostridium difficile* (*C. difficile*) infection, which has an incidence of around 1.8% as reported in a systematic review in 2017 [4]. This systematic review acknowledges a paucity of high level evidence but draws several conclusions, such as consideration to utilisation of preoperative probiotic and judicious antibiotic usage, avoiding proton pump inhibitors (PPIs) and avoiding ileostomy reversal delay periods to less than six months [4].

*C. difficile* infection can be a sinister disease process caused by a gram-positive anaerobic rod-shaped bacterium that reproduces via spores [4]. Whilst colonisation with *C. difficile* can be asymptomatic, symptomatic infections can result in a spectrum of symptoms, ranging from mild diarrhoea to severe complications, such as pseudomembranous colitis, toxic megacolon and death [5]. A number of risk factors have been identified for the development of *C. difficile* infection, including recent hospitalisation, broad-spectrum antibiotic use such as cephalosporins and penicillins and the use of PPIs. Further risk factors include a previous history of *C. difficile* infection, previous cytomegalovirus (CMV) infection, being on haemodialysis, having chronic renal disease or being on immunosuppressive agents or corticosteroids [5].

Concurrent *C. difficile* infection in patients undergoing an ileostomy reversal has been associated with significant morbidity. These infections usually present asymptomatic or as loose stools, which makes the diagnosis of *C. difficile* infection challenging due to loose stools being a potentially expected symptom post reversal of ileostomy [6]. When severe, the *C. difficile* infection then progresses to fulminant colitis or toxic megacolon, which have significant morbidity and mortality [7]. While there have been small retrospective/prospective studies and case reports regarding the development of *C. difficile* infection post ileostomy reversal, there have been no larger studies regarding the relationship, risk factors and associations between ileostomy reversal and development of *C. difficile* infection. Thus far, the only large study into *C. difficile* infection post ileostomy reversal using data from a Centers for Disease Control and Prevention (CDC) database, and as a result, the authors were unable to corelate any established risk factors for *C. difficile* infection to patients [8].

It is known that bowel surgery and formation of ileostomy can alter the gut microbiome, resulting in differences in amino acid distribution, pH and microbe concentrations in the gut [9]. This is potentially an explanation for the development of *C. difficile *infection post formation/reversal of ileostomy (especially in the disused section of colon downstream from the ileostomy) [4]. Thus far, no study has been published to investigate the role and impact of pre-operative *C. difficile* testing prior to the reversal of ileostomy. To further guide future research, the authors sought to perform an audit targeting potential risk factors for the development of *C. difficile* in the postoperative period in patients undergoing ileostomy reversal.

This article was previously presented as a poster at the 18th Congress of Asia Pacific Federation of Coloproctology (APFCP2021) on November 25, 2021.

## Materials and methods

This retrospective single-centre review was conducted within the General Surgery Department at Sir Charles Gairdner Hospital, a tertiary centre in Perth, Western Australia. All patients undergoing reversal of ileostomy were identified through operative records over a 2.5-year period (January 2019 to June 2021) and included in the retrospective audit. Data points including demographics and specific identified risk factors, such as a previous history of *C. difficile* infection and any antibiotic use (divided into preoperative, intraoperative and postoperative categories), were extracted from patient notes, focusing on the index admission when the ileostomy was formed. Patients that did not undergo *C. difficile* testing were assumed to be negative for *C. difficile* infection given their asymptomatic status not requiring *C. difficile* testing.

Statistical analysis

Statistical analysis was performed using IBM SPSS Statistics for Windows, version 27 (released 2020; IBM Corp., Armonk, New York, United States), with univariate analysis performed to test independent identified risk factors on the development of *C. difficile* infection. An alpha level of 0.05 was set and significance was calculated using Fisher’s exact test given the small sample size identified.

Ethics

As per the National Health and Medical Research Council's National Statement on Ethical Conduct in Human Research (May 1, 2022), the project was low risk and was approved by the hospital's Human Research Ethics Committee (Ref. # 42000).

## Results

A total of 69 patients who underwent ileostomy reversal were identified and included during the period of January 2019 to June 2021. The mean age of the patients was 57.2 years old (range 19-89, standard deviation (SD) 17.8). The underlying pathology in the cohort included cancer (n = 50), diverticulitis (n = 5), IBD (n = 9) and other benign pathologies (n = 5). The mean duration to ileostomy reversal was 332 days (range 13-2,984 days) with a mean postoperative admission of 16.1 days (range 2-49 days). Of the cohort, six patients (8.70%) tested positive for *C. difficile* during their admission for ileostomy reversal. Patient demographics and risk factors are summarized in Tables 1 and 2. Of note, two patients had significant complications related to *C. difficile* infection post reversal. One patient had non-resolving *C. difficile* infection and underwent a total proctocolectomy and end ileostomy. Another patient passed away one week post reversal from fulminant *C. difficile* colitis.

**Table 1:** Demographic characteristics of patients undergoing ileostomy reversal

n = 69	%	n
Gender		
Male	58%	40
Female	42%	29
Diabetic		
No diabetes	88.4%	61
Type II diabetes	11.6%	8
Smoking status		
Non-smoker	50.7%	35
Ex-smoker	34.8%	24
Current smoker	14.5%	10
Ileostomy type		
Diverting loop	91.3%	63
End ileostomy	8.7%	6
Underlying pathology		
Cancer	72.5%	50
Diverticulitis	7.2%	5
Inflammatory bowel disease	13%	9
Other (benign)	7.2%	5
Downstream anastomosis		
Stapled	73.9%	51
Sutured	18.8%	13
No anastomosis	7.2%	5
*C. difficile* testing post ileostomy reversal		
Yes	39.1%	27
No	60.9%	42
*C. difficile* positive on reversal		
Yes	8.7%	6
No	91.3%	63
Ileostomy reversal technique		
Stapled	5.8%	4
Sutured	94.2%	65

**Table 2:** Risk factors of patients undergoing ileostomy reversal CMV: cytomegalovirus

n = 69	%	n
Past *C. difficile* infection		
Yes	4.3%	3
No	95.7%	66
Past CMV infection		
Yes	2.9%	2
No	97.1%	67
Proton pump inhibitor or H_2_ receptor antagonist use		
Yes	58%	40
No	42%	29
Perioperative antibiotic use		
Preoperative	24.6%	17
Intraoperative	91.3%	63
Postoperative	56.5%	39
Chemotherapy use		
Yes	58%	40
No	42%	29
Immunosuppressant use		
Yes	11.6%	8
No	88.4%	61
Postoperative anastomotic leak or intra-abdominal collection		
Yes	26.1%	18
No	73.9%	51

The results of univariate analysis for antibiotic use are summarized in Table 3. Postoperative use of quinolone antibiotics was statistically associated with an increased risk of developing *C. difficile* on ileostomy reversal (odds ratio (OR) = 15.25, confidence interval (CI) 95%, p = 0.035). Intraoperative nitroimidazole use was statistically associated with a reduced risk of *C. difficile* infection on ileostomy reversal (OR = 0.16, CI 95%, p = 0.045).

**Table 3:** Odds ratio for C. difficile infection in the perioperative period (index operation where ileostomy was formed)

	Odds ratio (95% CI)	P value
Preoperative antibiotic use	3.50 (0.64-19.30)	0.154
Cephalosporin	0.91 (0.84-0.98)	0.567
Penicillin	5.30 (0.93-30.10)	0.076
Nitroimidazole	1.38 (0.14-13.30)	0.582
Cyclic lipopeptides	0.98 (0.95-1.00)	0.913
Bactrim	12.40 (0.67-229.40)	0.168
Intraoperative antibiotic use	0.43 (0.42-4.50)	0.433
Cephalosporin	0.19 (0.33-1.07)	0.076
Penicillin	7.38 (1.02-53.20)	0.081
Nitroimidazole	0.16 (0.026-0.94)	0.045
Lincosamide	0.98 (0.95-1.02)	0.913
Postoperative antibiotic use	1.60 (0.273-9.38)	0.690
Cephalosporin	0.83 (0.74-0.93)	0.338
Penicillin	2.85 (0.49-16.70)	0.221
Nitroimidazole	0.70 (0.08-6.50)	0.611
Lincosamide	0.98 (0.95-1.02)	0.913
Cyclic lipopeptides	0.98 (0.95-1.02)	0.913
Bactrim	0.98 (0.95-1.02)	0.913
Quinolone	15.25 (1.68 -138.41)	0.035
Glycopeptide	6.10 (0.47-79.52)	0.242
Macrolide	0.97 (0.93-1.01)	0.832
Carbapenem	2.95 (0.28-31.67)	0.359

Of the risk factors examined, none had statistically significant association with the development of *C. difficile* on ileostomy reversal, as shown in Table 4. However, of note is that patients who had underlying diverticulitis as the cause for the ileostomy formation (OR = 10), past CMV infection (OR = 12.4), being immunosuppressed (OR 4.75) or had a postoperative leak or collection (OR = 3.2) demonstrated higher risk for developing* C. difficile* post ileostomy reversal. Patients who underwent reversal of end ileotomies had a reduction in odds ratio in the development of *C. difficile* post reversal when compared to patients undergoing reversal of diverting ileostomies.

**Table 4:** Risk factors for C. difficile Infection IBD: inflammatory bowel disease, CMV: cytomegalovirus, PPI: proton pump inhibitor

Risk factor	Odds ratio	95% confidence interval	p-value
Female gender	1.286	0.4-4.130	0.501
Type II diabetes	0.286	0.073-1.117	0.140
Smoking	1.524	0.479-4.848	0.351
End ileostomy	0.476	0.066-3.439	0.433
Underlying cancer	0.340	0.062-1.680	0.203
Underlying diverticulitis	10	1.280-78.117	0.057
Underlying IBD	0.857	0.775-0.948	0.418
Past *C. difficile* infection	6.100	0.468-79.524	0.242
Past CMV infection	12.40	0.670-229.391	0.168
PPI or H_2_ antagonist use	0.703	0.131-3.759	0.499
Chemotherapy use	0.329	0.056-1.933	0.198
Immunosuppressant use	4.750	0.714-31.580	0.140
Post-operative leak/collection	3.200	0.583- 17.553	0.178
Ileostomy type: diverting	1.105	0.767-1.592	0.433
Ileostomy type: end	0.476	0.066-3.439	0.433

## Discussion

The development of *C. difficile* infection post ileostomy reversal, while uncommon, can lead to severe morbidity and mortality. The consideration into the role and impact of preoperative *C. difficile* testing led to this audit being performed.

The incidence of development of *C. difficile* infection post ileostomy reversal in our cohort of 8.7% was much higher than the previously reported rate of 1.8% [4]. A majority of the patient population analysed in the systematic review which quoted the incidence rates of 1.8% was derived from a 2013 matched cohort study involving a national United States of America Healthcare Cost database, with the incidence of development of *C. difficile* infection being reported at 1.6% (217 of 13,245 patients) [8]. Being a large population dataset, the study was unable to identify specific risk factors contributing to development of *C. difficile*, such as antibiotic use. We were also unable to fully compare demographics as these were briefly reported, although our demographics for age and gender were similar in nature. Of interest was that in their study patients managed in tertiary institutions demonstrated a higher propensity to the development of *C. difficile* infection, correlating to the results of our audit. This suggests that there may be institutional factors contributing to the higher incidence of *C. difficile* infection described in our study that have not been adequately identified. A potential explanation is that the patient cohort at a tertiary centre encompass more comorbid patients, putting them at higher risk of *C. difficile* infection.

It is also interesting to note that patients who underwent ileostomy formation had much increased risk of developing *C. difficile* infections if the primary underlying pathology was diverticulitis, with almost 10 times risk compared to patients undergoing the same procedure for IBD or cancer. Similarly, if the patient had a postoperative anastomotic leak during their index ileostomy formation their risk of *C. difficile* infection is much higher. This is presumably related to the use of antibiotic regimes in flares of diverticulitis and treatment for the anastomotic leaks, which ultimately result in changes to the intestinal microbiome, predisposing the patient to increased risk of *C. difficile* once intestinal continuity has been restored. This is also likely why patients with past *C. difficile* infection are also at higher risk of *C. difficile* infection post ileostomy reversal, as their intestinal tracts are likely colonised with the bacteria.

The use of intraoperative nitroimidazole (such as metronidazole) was associated with a reduced risk of developing *C. difficile* infection post ileostomy reversal. This could be attributed to reduced *C. difficile* colonisation in the disused colon with metronidazole use and higher likelihood of indigenous gut microbiome recolonisation. Fernandes et al. described significantly reduced rates of *C. difficile* infection in patients who received a single-dose metronidazole during induction compared to patients who received three doses of cefuroxime and metronidazole [10]. As of yet, no study has examined if a prophylactic course of metronidazole prior to ileostomy reversal would be beneficial. In addition, quinolone use (such as ciprofloxacin) demonstrated significantly elevated risk of *C. difficile* infection post reversal. This is not unexpected, as quinolone use has been long known to be linked to the development of *C. difficile* infection [11]. We recommend against quinolone usage in the perioperative period during ileostomy reversal.

The main weakness of the study is the small sample size. As a result, there were constraints in finding statistically significant relationship between the factors examined and the *C. difficile* infection post ileostomy reversal. Furthermore, most of patients examined did not have *C. difficile* testing prior to their ileostomy reversal. This was rectified with the uniform handling of missing data as described in the Methods section.

Based on the incidence of *C. difficile* infection post ileostomy reversal at our institution, a study population of 123 patients is required in a potential future study design to ensure that the study is adequately powered to within 5% of the population (p = 0.05). A consideration for future research can be a prospective blinded study to test all patients undergoing ileostomy reversal for the presence of *C. difficile* within the disused colon, and if these are eventually symptomatic post reversal (intention to treat analysis). For this study, consideration would also need to be given to asymptomatic *C. difficile* colonisation to the disused colon and a protocol designed to differentiate between symptomatic *C. difficile* infection or expected disuse colitis post ileostomy reversal. The timespan for this would be estimated around six years in our institution for the project to have sufficient patient recruitment to adequately power this study, although this would be considerably shortened if performed across multiple centres.

## Conclusions

*C. difficile* infection post ileostomy reversal can cause significant impact on a patient’s outcome in the postoperative period, and future directions in research can examine this in further detail, with focus on specific known risk factors, such as anastomotic leak in the index admission, previous *C. difficile* infection or having the underlying pathology be related to diverticulitis. In adddition, specific antibiotic usage, such as the nitroimidazole and quinolone groups (especially in the intra- and postoperative period during index ileostomy formation) can potentially have an impact on the development of *C. difficile* infection and their usage during these periods should be carefully considered. These data can assist in developing further recommendations and guidelines in the role of *C. difficile* testing prior to ileostomy reversal.

## References

[1] Bax TW, McNevin MS (2007). The value of diverting loop ileostomy on the high-risk colon and rectal anastomosis. Am J Surg.

[2] David E.Beck (2019). Intestinal Stomas. Gordon and Nivatvongs’ Principles and Practice of Surgery for the Colon, Rectum, and Anus (4th edition).

[3] Chow A, Tilney HS, Paraskeva P, Jeyarajah S, Zacharakis E, Purkayastha S (2009). The morbidity surrounding reversal of defunctioning ileostomies: a systematic review of 48 studies including 6,107 cases. Int J Colorectal Dis.

[4] Harries RL, Ansell J, Codd RJ, Williams GL (2017). A systematic review of Clostridium difficile infection following reversal of ileostomy. Colorectal Dis.

[5] Hung YP, Lee JC, Lin HJ, Liu HC, Wu YH, Tsai PJ, Ko WC (2015). Clinical impact of Clostridium difficile colonization. J Microbiol Immunol Infect.

[6] Swathi G, Y. S. H, Reddy JPN, Meesala L (2019). A surgical audit on ileostomy - from creation to closure. Journal of evidence based medicine and healthcare.

[7] Li M, Behrenbruch C, Wong A, An V (2020). Megacolon following ileostomy reversal due to Clostridium difficile. ANZ J Surg.

[8] Wilson MZ, Hollenbeak CS, Stewart DB (2013). Impact of Clostridium difficile colitis following closure of a diverting loop ileostomy: results of a matched cohort study. Colorectal Dis.

[9] Ohigashi S, Sudo K, Kobayashi D, Takahashi T, Nomoto K, Onodera H (2013). Significant changes in the intestinal environment after surgery in patients with colorectal cancer. J Gastrointest Surg.

[10] Fernandes R, Robinson P, Rangarajan K, Scott S, Angco L (2017). The role of single-shot metronidazole in the prevention of Clostridium difficile infection following ileostomy reversal surgery. Int J Colorectal Dis.

[11] Deshpande A, Pant C, Jain A, Fraser TG, Rolston DD (2008). Do fluoroquinolones predispose patients to Clostridium difficile associated disease? A review of the evidence. Curr Med Res Opin.

